# Seven features of safety in maternity units: a framework based on multisite ethnography and stakeholder consultation

**DOI:** 10.1136/bmjqs-2020-010988

**Published:** 2020-09-25

**Authors:** Elisa Giulia Liberati, Carolyn Tarrant, Janet Willars, Tim Draycott, Cathy Winter, Karolina Kuberska, Alexis Paton, Sonja Marjanovic, Brandi Leach, Catherine Lichten, Lucy Hocking, Sarah Ball, Mary Dixon-Woods, the SCALING Authorship Group, Cathy Bevens

**Affiliations:** 1 THIS Institute (The Healthcare Improvement Studies Institute), Department of Public Health and Primary Care, University of Cambridge, Cambridge, Cambridgeshire, UK; 2 Department of Health Sciences, University of Leicester, Leicester, Leicestershire, UK; 3 Department of Translational Health Sciences, University of Bristol, Bristol, UK; 4 PROMPT Maternity Foundation, Bristol, UK; 5 Department of Sociology and Policy, Aston Medical School, Aston University, Birmingham, UK; 6 RAND Europe, Cambridge, Cambridgeshire, UK

**Keywords:** qualitative research, healthcare quality improvement, patient safety, obstetrics and gynecology

## Abstract

**Background:**

Reducing avoidable harm in maternity services is a priority globally. As well as learning from mistakes, it is important to produce rigorous descriptions of ‘what good looks like’.

**Objective:**

We aimed to characterise features of safety in maternity units and to generate a plain language framework that could be used to guide learning and improvement.

**Methods:**

We conducted a multisite ethnography involving 401 hours of non-participant observations 33 semistructured interviews with staff across six maternity units, and a stakeholder consultation involving 65 semistructured telephone interviews and one focus group.

**Results:**

We identified seven features of safety in maternity units and summarised them into a framework, named *For Us* (For Unit Safety). The features include: (1) commitment to safety and improvement at all levels, with everyone involved; (2) technical competence, supported by formal training and informal learning; (3) teamwork, cooperation and positive working relationships; (4) constant reinforcing of safe, ethical and respectful behaviours; (5) multiple problem-sensing systems, used as basis of action; (6) systems and processes designed for safety, and regularly reviewed and optimised; (7) effective coordination and ability to mobilise quickly. These features appear to have a synergistic character, such that each feature is necessary but not sufficient on its own: the features operate in concert through multiple forms of feedback and amplification.

**Conclusions:**

This large qualitative study has enabled the generation of a new plain language framework—For Us—that identifies the behaviours and practices that appear to be features of safe care in hospital-based maternity units.

## Introduction

Maternity care is generally safe in high-income countries but is not consistently safe.^1^ As in other areas of clinical practice, it demonstrates a pattern of unwarranted variation in both practices and outcomes.^1–4^ Avoidable harm in childbirth can have devastating consequences for families,^5 6^ and is an increasingly important driver of cost pressures in health systems through claims for negligence/malpractice.^1 7 8^ Poor care is increasingly recognised as a threat to human rights and the women’s and children’s health agenda.^9^ Improving maternity care is thus a worldwide imperative.

The dominant approach thus far has focused on identifying what goes wrong in maternity care. This work has offered important insights into factors that contribute to poor outcomes, including failure to recognise and escalate problems, lack of psychological safety, inadequate leadership, issues with service capacity and staff turnover, and poor communication and teamwork.^5 10–15^ All are vividly evident in recent reports of catastrophic degradations of maternity care.^16 17^ Despite the large body of evidence that has emerged from scandals and adverse events in maternity care, progress in improving safety has remained frustratingly slow. There may be considerable value in a turn to a more positive approach that seeks to identify what it is that characterises units that perform well, even though they appear to operate under similar conditions to those that underperform.^18^

The search for the features of exceptionally high-performing settings is now a uniting trait of several literatures, including those on high reliability organisations,^19–21^ safety engineering, safety II and resilient healthcare,^22–25^ and positive deviance approaches.^26–28^ Although they have emerged from somewhat different theoretical perspectives and have varying emphases, these literatures share several conceptual similarities.^26^

A consistent finding is the forces that create positive conditions for safety may be at least partially invisible to those who create them because they remain tacit or habitualised: they require structured study to surface them. Indeed, a key principle emerging from the literature on positive deviance is that the ability to solve a problem may already exist within the community experiencing the problem, and the challenge is to find and share particular practices or solutions already in use that may be of benefit to all.^27–29^ A further growing insight of recent work, particularly in the area of positive deviance, is that, to provide useful and actionable guidance for clinicians and managers, it may be especially important to produce descriptions of ‘what good looks like’ that are specific to their particular areas of care, rather than operating at the level of generality.^26–29^

An explicit, empirically based account is therefore likely to be valuable in identifying features of safety that are important for maternity units. Also important is ensuring that the findings are available, useful and accessible for those who might seek to use them as a basis for reflection and action. In previous work,^18^ we produced a theoretically informed characterisation of key features associated with safety in maternity care through an ethnographic study of a single high-performing maternity unit (table 1). In the new study we report here, we aimed to deepen, mature and refine our understanding of these features through an extended study in a larger sample of units and to produce a plain language framework that would be practically useful for those working in maternity care in promoting understanding of ‘what good looks like’.

**Table 1:** Features of safety identified in initial study of a single high-performing maternity unit (adapted from Liberati et al, 2019)^18^

Collective competence	The unit displays a sense of interdependency. Collegial behaviours and strong social ties are visible among staff. Care is organised around the shared goal of safe childbirth. Professional boundaries are managed flexibly with deference to expertise rather than hierarchy.
Insistence on technical proficiency	Very high standards of proficiency in clinical tasks are expected. High-fidelity structured training is combined with informal learning through mentoring and legitimate peripheral participation.
Monitoring, coordination and distributed cognition	Mechanisms are in place to maintain a shared awareness of the external situation in the maternity unit. Staff in coordinating roles play a control room function.
Clearly articulated and constantly reinforced standards of practice, behaviour and ethics	Values of ethical and safe behaviour are clear, articulated and reinforced through role modelling. Staff make positive use of social control mechanisms to ensure that other people behave in a way that is aligned with the unit’s standards.
Monitoring multiple sources of intelligence about the unit’s state of safety	Many forms of data are used to sense problems. Routine clinical data are scrutinised, updated and made available to all staff. Soft intelligence (such as patient feedback and staff ground knowledge) is used to learn and improve safety and nurtured through a widespread sense of psychological safety.
Highly intentional approach to safety and improvement	Commitment to safety is collectively pursued and socially legitimised (not externally imposed). Combination of formal risk management (ie, allocated roles and formal activities) and embedded risk management (frontline clinicians preparing for risky situations and detecting small signs of deterioration).

## Methods

We conducted a two-part study: a multisite ethnography involving the initial high-performing index site and five further sites to characterise the features of safety in maternity units and a large-scale stakeholder consultation to inform production of a plain language framework describing these features.

### Multisite ethnography

We undertook non-participant ethnographic observations^30 31^ and conducted semistructured interviews in six UK maternity units in two phases (table 2). The first phase involved intensive study of the index unit (site 1), which was selected as a high performing site on the basis of its rates of birth complications (one of the lowest in the UK),^32^ sustained improvements on a range of perinatal outcome measures^18 32 33^ and staff survey results indicating positive teamwork and safety culture and high job satisfaction.^34^ The unit counted approximately 6500 births per year and had a midwife-to-birth ratio of 1:30. Site 1 developed the PRactical Obstetric Multi-Professional Training (PROMPT) programme in 2000^32 35^ and has continuously implemented and updated its local programme ever since. The ethnographic data at the index site were collected in December 2014, March 2015 and July–August 2017.

**Table 2:** Ethnographic data collected in six maternity units

Ethnographic research phase	Phase 1	Phase 2				
Aim	Produce a comprehensive account of the mechanisms underlying the safety outcomes seen in the index maternity unit.	Evaluate the extent to which the mechanisms underlying safety at the index site were also visible in a sample of maternity units exposed to PROMPT and to refine and develop a deeper understanding of these mechanisms.				
Site	Index maternity unit (site 1)	Site 2	Site 3	Site 4	Site 5	Site 6
Observations	143 hours	40 hours	64 hours	52 hours	34 hours	68 hours
Interviews with staff members	12	4	6	4	3	4
Data collection timeline	December 2014, March 2015, July–August 2017	September 2017	September–October 2017	October–November 2017	January 2018	May–June 2018
Total	401 hours of ethnographic observations 33 semistructured interviews					

PROMPT, PRactical Obstetric Multi-Professional Training.

The second phase involved five additional units (sites 2–6), which were selected because they had all taken part in the PROMPT Train the Trainer programme developed by site 1.

Since this sample had all been exposed to the same improvement intervention, we anticipated that some of the features observed in site 1 might also be present in these units and that further investigation would allow them to be better understood. Though these sites were not selected on the basis of performance, their implementation of PROMPT was variable, indicating that they were likely to demonstrate some differences from one another, and cross-site comparison would therefore be facilitated. The five units were based in urban locations and included three medium-sized units (between 3000 and 5000 births per year) and two large units (over 5000 births).

Ethnographic data collection in these five units took place between September 2017 and June 2018 and included: (1) observations of routine care activities in maternity units (labour wards, birth centres, operating theatres, and antenatal and postnatal wards), including observation of briefings, handovers and training events; (2) semistructured interviews with doctors, midwives and members of the managerial and risk management teams; (3) and informal conversations between the researchers and healthcare professionals.

Data were collected by three researchers (JW, EGL, CT), all social scientists. Throughout the ethnography, our sampling strategy for interviews and informal conversation was informed by the principle of maximum variability, meaning that we endeavoured to access as many different points of views as possible (in terms of role, discipline and seniority). We spent more time in the index site than in sites 2–6, given the different aims of each of the two study phases (table 2). The amount of time spent on each site was also determined by the principle of ‘information power’,^36^ the degree of the variability captured by researchers and feasibility constraints such as availability of units to host researchers.

Analysis of the observational and interview data from the six sites was conducted using a systematic and iterative approach based on the constant comparative method.^37^ First, as previously reported, we analysed data collected in site 1 using an inductive, open coding approach, progressively organising codes into broader themes that were theoretically guided by previous literatures.^18^ We constantly compared interview and observational data to enhance our understanding of what we observed in the field and to capture experiences and practices of participants. We ultimately developed a theoretically informed account of the features of a very safe maternity unit (table 1) organised into a framework.

This framework was used as a set of sensitising constructs^37^ for the analysis of the data from sites 2–6, with the aim of refining and deepening understanding of the features originally identified in site 1. Data from each unit was processed individually, allowing identification of the extent to which particular features from the site 1 framework were present, and then we compared across units to develop a synthesised account of each feature. Consistent with the constant comparative method, the categories were modified iteratively in response to the data, resulting in respecification of some categories and/or new categories being developed.

### Stakeholder consultation

We conducted 65 semistructured interviews (September 2018–February 2019) and one focus group (September 2019) with the aim of enhancing the learning from the ethnographic study and using it to develop a concise, plain language framework to summarise the features of safe maternity units in an accessible and meaningful way. A first iteration of the framework, based on the findings from both phases of the ethnographic study, was used in interviews with participants. Participants were asked to comment on categories, comprehensiveness and relevance, clarity of terminology and language in this draft version.

Individuals were sampled purposefully to cover a diversity of professional roles, affiliations and perspectives on maternity care, including those of users of maternity services. Our sample included seven stakeholder groups (table 3). We aimed to speak with 5–10 individuals per stakeholder group, expanding our sampling if more variability arose within each group; this principle determined the final number of interviewees. None of the interviewees worked in sites 1–6. The response rate to interview invitations was 54%.

**Table 3:** Stakeholder consultation sample

Stakeholder group	Number of individuals interviewed	Number of individuals who took part in the focus group
Women who had recently used maternity services	8	
Frontline clinicians (doctors and midwives)	19	4
Middle managers (heads of midwifery and clinical directors)	6	
Individuals with a leading role in maternity programmes or initiatives	8	2
Improvement experts	11	
Policy makers (eg, from National Health Service organisations)	8	2
Members of relevant professional bodies (Royal Colleges of Obstetricians and Gynaecologists and Royal College of Midwives)	5	
Total	65	8

The interview transcripts were analysed deductively, based on the structure of the topic guide, and organised by the pragmatic objective of refining the framework we had developed from the earlier analyses. Once a revised version of the framework was developed, it was tested against our original ethnographic data to ensure consistency and further optimised through feedback from a focus group comprising three stakeholder groups (table 3). Online supplemental file 1 shows how the framework evolved over time through our iterative consultation.

10.1136/bmjqs-2020-010988.supp1 Supplementary data

### Ethics approval

Health Research Authority research ethics committee approval was obtained for the study, as well as site-specific approvals for each unit. All individuals we interviewed signed a consent form; we obtained oral permission for observations.

## Results

Across the six sites in the ethnographic study, we conducted 401 hours of observations and 33 interviews (table 2). None of the five new units (sites 2–6) demonstrated all six of the features originally seen in our original analysis of the index site (table 1), but many of the features were visible in varying forms and degrees across the units. The analysis of these new data generated a deeper and more mature characterisation of the features of safety and enabled their clearer description and articulation, for example, some features were renamed, some mechanisms were regrouped under different headings and other mechanisms were reconfigured within features. Our synthesis of learning across the sites, together with our stakeholder consultation, resulted in a plain language framework with seven features (table 4). Six of these features were, broadly speaking, modifications of those that were reported in the original six-feature analysis based on site 1 only, while one further feature (on systems and processes designed for safety, row six in table 4) gave more prominence to mechanisms that had been more implicit in our previous analysis.

**Table 4:** For Us framework

Features	Description and examples
Commitment to safety and improvement at all levels, with everyone involved	The unit shows an authentic commitment to learning from risky situations and adverse events, and it uses this learning to drive improvements. Staff are skilled in noticing hazards and seek to address them in real-time. When appropriate, hazards are reported so that the whole unit can learn. Staff invest in making the unit better. They are always looking for ways to improve working processes and the care environment—often through small-scale, easily actionable ideas—and are praised for their efforts. Individuals in management roles are visible and accessible. They listen carefully to frontline staff and families, seeking to respond promptly to concerns or suggestions reported to them. The unit has a range of formal risk management systems, processes and roles (including audits and/or a risk management team) that are known, trusted and used by staff in the unit.
Technical competence, supported by formal training and informal learning	Individuals are expected to perform their clinical tasks to a high standard of proficiency. The unit invests in keeping staff trained and up to date. Regular high-quality training sessions are mandatory for all members of staff, and the unit management ensures that everyone has allocated time to attend. Training is usually multidisciplinary and includes structured teaching, skill drills and simulations. People also learn in less formal ways, for example, through mentorship, observing colleagues at work and discussing and reflecting on clinical cases. Senior members of staff make sure that more junior staff have opportunities to debrief and ask questions after experiencing complex clinical situations and that they learn from theirs and others’ experience. A social space is accessible to all staff (a communal coffee room, for example) to support informal knowledge sharing, real-time information updates and reflection. The many different forms of learning allow staff to demonstrate competence, confidence and coordination in high-stress, risky situations and help to create trust among team members.
Teamwork, cooperation and positive working relationships	Teamwork is central to all of the activities carried out in the unit. Care, training and research are conducted with the input of all professions and disciplines. People in different roles respect each other and value everyone’s contributions to achieving the goals of the unit and upholding its values. Through working and training together, people are aware of each other’s roles, skills and competencies (who does what, how, why and when) and can work effectively together, thus demonstrating ‘collective competence’. When deciding who should perform a certain task, the team regard skills and experience as more important than seniority or professional roles: the person with the right skills for the specific task will intervene. When disagreements happen between professions or roles (eg, on treatment decisions), they are settled calmly through open, thoughtful discussion and through reference to shared goals. People do not resort to hierarchies, displays of power, or aggressive behaviour. People look after each other. Relationships are good, and any disruptive or bullying behaviours are recognised and managed effectively. Staff well-being and morale are recognised as important contributors to safety.
Constant reinforcing of safe, ethical and respectful behaviours	The goals and values of the unit are clear: achieving good birth outcomes and promoting the dignity and well-being of parents and families. There is a shared expectation that members of staff will behave consistently with these goals and values. Expected standards of practice are reinforced through the behaviours of everyone in the unit, including all professions and individuals at all levels—from the most junior to the most senior. Newcomers are supported to understand and adhere to the unit’s high standards but are also encouraged to make suggestions for improvement based on previous experience. People intervene if the goals and values of the unit are not upheld. They do so mostly in informal ways (eg, by using humour or having a ‘private word’) but are ready to intervene more formally when needed (eg, through reporting systems and escalating). Unsafe or inappropriate behaviours are noticed and corrected in real time, so they do not become normalised. Although the highest standards of practice are expected, it is recognised that errors will sometimes happen. Errors are recognised both as problems and as opportunities for learning. People are encouraged to discuss them openly, and actions are taken to reduce risk of their recurrence.
Multiple problem-sensing systems, used as basis of action	The unit uses multiple methods to ‘sense’ and anticipate problems and identify opportunities for improvement, including staff and families’ voice, hard data and clinical simulation. These multiple forms of intelligence are also used to identify good practices and celebrate them where appropriate. Families are encouraged to share their experience, in real time and retrospectively, through formal and informal feedback systems. This feedback is seen as key for improving care. Members of staff feel that they can speak up for safety. They are confident that their concerns will be heard and that action will be taken as a result, whenever possible. This sense of psychological safety cultivated on the unit makes it possible to learn from everyday events. Clinically relevant data are collected and constantly monitored using visual methods (a clinical dashboard, for example) to identify concerning trends and guide improvement efforts. Members of staff are reminded about the importance of looking at and interrogating data.
Systems and processes designed for safety and regularly reviewed and optimised	Working processes and information technology are well designed, and kept functional and up to date. The unit’s equipment and the physical environment are designed consistent with human factors and ergonomics principles to be safe, appropriate and easy to use. People constantly review and seek to optimise working processes (eg, operating theatre scheduling) and tools (eg, postpartum haemorrhage kits) to meet the requirements of excellent care provision. Simulation is used to observe how systems and processes operate in realistic conditions and to test the usability and appropriateness of equipment and other resources needed for care. Once good practice is identified, it is standardised and spread across the unit to avoid unwarranted variation.
Effective coordination and ability to mobilise quickly	Well-functioning systems (eg, IT systems and whiteboards) are in place to capture and share up-to-date information regarding each woman. These systems help to identify risks early and to initiate an effective response. Structured handovers and regular safety huddles, ward rounds and board rounds enable a shared, helicopter-level understanding of the state of the unit as a whole in real time. Identified individuals in the team have specific responsibility and expertise for patient flow and management between the different care settings. Mandatory training emphasises the importance of situational awareness, which includes enabling staff to recognise the important elements of their environment that may affect patient care. Simulation-based training and structured emergency protocols allow staff to be competent and confident in responding to crises.

### Commitment to safety and improvement at all levels, with everyone involved

In our original study in site 1, we identified a ‘highly intentional approach to safety and improvement’, involving a commitment to safety that was collectively pursued, as a feature of safety in maternity units.

[When we notice delays in inductions], we all, as a team, think ‘This is not the care we want to give, why is this happening, what can we do about it?’ I think we – the consultants, the midwives, the risk management, the managers – we know what we should be doing (…) we always try and come up with ideas to make improvements. (Ethnography, interview with senior midwife, site 1)

Our new analysis, based on all six sites, deepened understanding of this feature of maternity safety (table 4). We found that, where present, it involved an authentic commitment to safety and improvement shared by all organisational members, embedded in the unit’s ethos and nurtured by people’s genuine desire to improve care even when faced with severe resource constraints. Robust risk management processes, systems and roles were known to, and trusted by, all members of staff. Staff constantly sought to make the unit better, both through learning from risky situations or adverse events, and through testing small-scale improvement ideas generated by those at the frontline of care. Achievements were celebrated but were not a source of complacency.^38^ Instead, a fertile state of what is known in the high reliability literature as ‘chronic unease’^39^ was demonstrated.

We’re constantly endeavouring to try and make it safer. And there’s always room for improvement. (Ethnography, interview with consultant anaesthetist, site 3)

I think we deal with problems as they arise. (…) If something does go wrong, we’re very quickly looking into it, it’s not a blame culture, it’s just to try and find out what went wrong and how we can do it better next time and whether lessons can be learned. (Ethnography, interview with midwife, site 3)

Where the drive towards improvement was not as visible in sites, safety policies and protocols were somewhat dislocated from routine practice. Frontline staff still reported potential risks but appeared less engaged with identifying possible solutions or opportunities for organisational learning. Shortage of time or resources, lack of proactive risk management systems or a sharp divide between frontline and management staff appeared to interfere with a commitment to safety.

But are defects learning opportunities here? I wouldn’t necessarily say so. No. (Ethnography, interview with registrar, site 4)

### Technical competence, supported by formal training and informal learning

In our original analysis, we identified an insistence on technical proficiency as a key feature of maternity unit safety. Our new analysis allowed further characterisation of this feature. When present, staff could perform their tasks to a very high standard of competence, and the importance of keeping skills and knowledge up-to-date was consistently reinforced supported by structured training that was mandatory for all and by protected time for attendance. Usually conducted in multidisciplinary settings, such training included teaching, skill drills and simulations and was recognised as an opportunity to strengthen team members’ understanding of their work environment and mutual roles as well as their technical skills.

They refreshed the use of protocols and algorithms, they used the actual trolley, and there was a lot of focus on technical skills. In the debrief after the scenario, they first talked about who in the team needs to be called when a PPH (post-partum haemorrhage) happens, how to assess the volume of blood, how to make sure that the communication with the blood test centre is handled properly. There were strong links between practice and training. They then talked at length about the communication and teamwork side of things. (Ethnographic observation of PROMPT training day, site 2)

Each group of participants were split into three further smaller groups. And they all had a go at doing the adult life on the patient dummy. While they were doing it, the trainer was correcting them in real life. So, if the chest compressions were not deep enough, she would say, you need to go deeper. And so, they had the possibility to self-correct while actually doing the training. (Ethnographic observation of PROMPT training day, site 3)

Learning and reflection was also embedded in routine practice. Essential to this was shared social spaces (eg, staffrooms with seating) where colleagues could spontaneously debrief after complex clinical situations. More experienced individuals also socialised juniors or peers through role modelling and mentoring.

Throughout the day, there was a lot of very brief and informal one-to-one teaching going on in the communal coffee room. Somebody will say to somebody ‘Have you seen this? Do you know what you do in this sort of situation?’ And this goes on between the midwives and the student midwives and the more newly qualified midwives. It also happens between the registrars and the SHOs [senior house officer] and the consultants. Everybody’s checking up on the learning of everybody else. (Ethnographic observation in labour ward, site 1)

Staff are really good at trying to support you with your training… So, if you say to [a senior midwife] in the morning that you want to try and do this competency today, (…) then they come and supervise you doing it. (Ethnography, interview with band 5 midwife, site 6)

There are lots of very newly qualified midwives and the really important path for their development is being supported by competent members of staff (…) A mentoring sort of atmosphere (…). Formal training is not the only learning modality. (Stakeholder consultation, interview with consultant obstetrician and gynaecologist)

Where this feature was visible, the effects of formal and informal training were evident in staff confidence, readiness and proficiency when undertaking complex procedures. Where fewer opportunities to learn from one another were available (often due to capacity pressures in the unit), staff competence was inclined to suffer and standards of good and safe practice might remain unclear to some team members (especially newcomers), resulting in individual confusion and collective unwarranted variation.

The busier [the unit] gets the less you are training up. (…) The co-ordinator, rather than watching a new midwife suture somebody’s perineum, which takes three times as long, ends up doing it [her]self or getting the doctor to come in and do it because you haven’t got time to stand there and watch. (Stakeholder consultation, interview with consultant obstetrician)

### Teamwork, cooperation and positive working relationships

While teamwork, cooperation and positive working relationships were elements of the features we described in our original analysis of site 1 (most obviously but not exclusively in the feature originally named ‘collective competence’), our extended analysis identified that they should be highlighted as a key feature of safety in maternity units. Where this feature was visible in our analysis, staff knew each other well, had good relationships and could work effectively across professional boundaries. An explicit emphasis on shared goals helped in managing the inevitable divergences of views that routinely occur between staff (eg, over clinical management) through respectful and open dialogue, rather than resorting to displays of brute power. Typically, this feature was associated with flexible hierarchies, including an expectation that the person with the right skills (not necessarily the most seniority or a particular professional role) would take the actions needed for high-quality clinical care.

There’s a really good relationship between the doctors and the midwives and as someone that’s new to this [hospital], I definitely felt very welcomed and I felt that the midwives were very keen to get to know me, to talk to me, to show me where things are (…). Everyone really cares for their women and is working towards helping them and achieving the best outcome. (Ethnography, interview with registrar, site 3)

It was an emergency C-section and the baby was not crying, so the paediatrician rubbed its back to get [baby] to cry but [the senior midwife] thought he was doing it too gently, so she stepped in and did it successfully. She said ‘Sometimes you just do what you have to do, you just switch into automatic mode to make sure things are safe’. (Ethnographic observation, site 6)

Where this feature was present, teamwork was paramount: people were aware of each other’s roles and skills, demonstrating a high degree of collective competence.^40^ Positive working relationships were also observed in spontaneous attempts to help each other and support members of the team who might be experiencing personal of professional difficulties. Well-being and morale were recognised as important contributors to safety.

[A] midwife told me about when she was having to resuscitate a baby, and she was in the room and the resuscitation wasn’t really going very well. And she turned to her side, just as she was starting to think, ‘Actually I need a different mask to be able to resuscitate this baby, a specialised mask’, and the other midwife was there, standing with the mask. She’d had the same thought, and it was there, ready and waiting for her, in her hands. She said it was almost like she’d read her mind. (Ethnographic observation on labour ward, site 5)

I think everybody cares. (…) I think we have got a very caring workforce. We've been very short of staff, midwives and doctors, but because we care about each other, we give that extra mile (…). I think generally people enjoy coming to work because everybody feels a bit like a family. (Ethnography, interview with senior midwife, site 1)

Conversely, when this feature was absent, higher levels of conflict and distrust between professional groups, and a lack of investment in multidisciplinary working, was evident. High staff turnover rates were also a particular challenge, hindering team members’ ability to form stable relationships, mutual trust and shared expectations on each other’s role and skills.

(In) poorly performing and failing maternity units… the first thing you always come across is very poor relationships, particularly between the staff groups (…) midwives and doctors at each other’s throats. (Stakeholder consultation, interview with consultant anaesthetist)

### Constant reinforcing of safe, ethical and respectful behaviours

In our original site 1 analysis, we found that an important characteristic of safety on maternity units involved clearly articulated and constantly reinforced standards of practice, behaviour and ethics. Our extended analysis confirmed the salience of this feature while also modifying our understanding of it somewhat (eg, shifting to a more specific emphasis on a culture of openness and collective learning). Where this feature was present, staff had sound understanding of the behaviours and conditions that promote safety and sought to make them clear to everyone in the team. Rather than being showcased as abstract mission statements, expected behaviours were articulated and reinforced on a daily basis through role modelling and through leading by example. Newcomers were supported to understand and adhere to high standards but were also encouraged to make suggestions for improvement.

Conversations [during the ward round] were thorough and calm. They were led by the consultant but everyone was asked for their input and opinion. (…) Once in the room, families were always included in the conversations, the consultant always asked the women for their opinion and if they had any questions. It felt like excellent role modelling by the consultant. (Ethnographic observation in labour ward, site 6)

Role-modelling (…), having more senior people around to talk to and share their experiences with, I think is a really important part of that knowing how to behave, knowing what’s acceptable and what’s not. (Stakeholder consultation, interview with registrar)

When people noticed disruptive or transgressive behaviours, they intervened so that unsafe practice did not become normalised. They did so mostly informally, having a private word, offering support and sometimes relying on humour but were willing to resort to more formal intervention (eg, reporting or escalating concerns) if needed.

[This] is really important because… the behaviour we tolerate is the behaviour we promote… (Stakeholder consultation, interview with senior midwife)

And actually I’d had my management questioned [by a midwife] (…) not in a derogatory way in front of a patient (…) more like in a, ‘Oh, why did that end up like that?’ (…) And I think that’s really good because actually if you’re concerned about a patient and I’m new and you don’t know anything about me, you should question it; and then I’m more than happy to justify it, because if I couldn’t then I’m doing the wrong thing, aren’t I? (Ethnography, interview with obstetric registrar, site 4)

Reinforcing expected behavioural standards meant that people had a clear sense of what was expected of them; without these shared expectations, mismatches between ‘work as imagined’ and ‘work as done’^41^ might go unnoticed, and opportunities to improve practice and avert risks may be lost. Shared values and norms also meant that, when errors did happen, they were seen as an opportunity for collective learning and discussed openly and honestly.

There was a senior medical colleague who was appearing quite stressed on the delivery suite… it felt a bit as if we were not going to be managing our day very well. (…) So I… spoke to another medical colleague and said ‘I think there’s a requirement for a bit of support up there today’. (…) I think it’s about having the ability as a team to recognise in each other… that we need help, and not being frightened to say “I think you need a bit of support today”. (Ethnography, interview with midwife, site 1)

### Multiple problem-sensing systems used as basis of action

In our original analysis, we identified the importance of monitoring multiple sources of intelligence as a feature of maternity unit safety. Our extended analysis reaffirmed this while further emphasising the value of using multiple problem-sensing systems as a basis of action. This feature was visible when staff gathered insights and knowledge from multiple sources to continuously learn and improve, and those in management roles acknowledged the importance of listening to those at the frontline of care. Members of staff and families accessing the service were actively encouraged to share ideas and concerns, and their experience was used to sense problems, self-assess and learn.

We’ve got the maternity services Facebook page (…) which is a really good source of getting feedback, because like that’s how people communicate now… (through) social media. (…) There’s [two] midwives that… make sure that they see those messages. (Ethnography, interview with midwife, site 3)

Critical to harnessing soft intelligence^42^ was the nurturing of a psychologically safe environment, where people felt they could share ideas and concerns, that they would be taken seriously and that action will be taken as a result. To this end, visible and responsive senior and management staff had a key role, and where it was absent the effects were stifling.

I walk the floor every morning. I walk to every area, and spend time, and so every day, (…) people would know… I would crop up at some point. So, [it’s easy for people] to say to me… that they noticed something or they’ve had an idea. (Ethnography, interview with head of midwifery, site 1)

I’ve multiple times taken cases that I’ve struggled with or I’ve reflected upon and thought ‘Did I manage that well or did I not manage that well?’, I’ve taken it to the morning meeting or personally to a consultant and said, ‘Can you have a look at this, can we discuss this, what do you think I did well here […or] badly here?’ (Ethnography, interview with obstetric registrar, site 4)

I think (…) [staff] are not slow in saying ‘Well actually there’s problems at the moment, the staffing is terrible’, they will highlight that. And risk management are absolutely supportive if the staffing isn’t adequate. Because unless it’s registered we can’t do anything about it and that’s very much our ethos for everything. (Ethnography, interview with senior midwife, site 1)

Use of hard data was also a crucially important mechanism. Where present, it involved close monitoring of clinically relevant data (eg, through maternity dashboards and incident reporting systems) and was used to identify concerning trends and areas for improvement. A shared awareness of the challenges of using data in a meaningful way (even when they were reliably collected) meant that every opportunity was taken to make data accessible. Aware of the challenges of using data in a meaningful way, safe units seek every opportunity to make data more accessible to frontline clinicians and central to their daily practice. Data were, for example, discussed on training days or used to conduct locally relevant audits.

You’ve got [to use] data well. There’s so much data that’s collected within maternity units. (…) One of the weaknesses of maternity is actually acting on it. (Stakeholder consultation, interview with consultant anaesthetist)

### Systems and processes designed for safety and regularly reviewed and optimised

In our original analysis, we found that optimised systems and processes were important, but we did not identify it as a standalone feature of maternity unit safety. Our extended analysis identified it as a key feature to be highlighted separately. Where this feature was present, system and processes and physical environment were continually improved to ensure usability and operational fitness. Where systems and processes were optimised, the equipment and information technology were purposefully designed to be safe and easy to use. For example, consistent with human factors and ergonomics principles, algorithms, emergency boxes or trolleys and stickers were used and regularly updated to support consistent practice and to ‘make the right way the easy way’ (ethnography, interview with consultant obstetrician, site 1). Training days—particularly simulation—were used as a forum to test the usability and appropriateness of the units’ resources and to optimise and update them when needed. Once good practice was identified, it was standardised and spread across the unit in order to minimise variation.

Sometimes by doing the drills, that’s when you identify problems, as well. So quite often when you’re running these scenarios, somebody will say, ‘Oh, well, I tried … to ring the such-and-such number’, or, ‘Well, we haven’t got that type of oxygen mask on community’, or – you know, it’ll be little things that come out as part of that training. And you say ‘Well, you should have a non-re-breathe mask in your kit’, or, ‘You should have this particular type of catheter for bladder-filling’, or… And it’s always the opportunity to find out if something (…) needs to be addressed. (Ethnography, interview with manager, site 1)

Conversely, unwarranted variation in process, systems, and equipment imposed substantial cognitive load on frontline staff. These defects in equipment supply distracted attention from risk anticipation and problem sensing, strained collective competence and increased wear and tear on staff. Examples of these faults were especially visible where mechanisms to ensure operational fitness were lacking.

They didn’t seem to do [the training] as if it was on the ward. The tools and things that they needed [for the drill stations] were ready for them in the rooms, rather than people having to go and look for them. So they weren’t really testing the systems that they had present on unit. It was more that they were testing that people knew what they needed to do, rather than what the systems were. (Ethnographic observation of training day, site 5)

[Speaking about a new online patient record system]. You could have your notes done within 20 minutes, 15 minutes when you were [physically] writing them. It’s sometimes two, two and a half hours now. (…) I think it’s taking time away from the patient. And sometimes you’re typing it all in, and it’s gone, probably ‘cause the wireless has gone off at that point […]. It’s very frustrating. (Ethnography, interview with midwife, site 5)

### Effective coordination and ability to mobilise quickly

In the original analysis, we identified monitoring, coordination and distributed cognition as a key feature. Our new analysis reaffirmed the importance of this feature of safety. Where present, it was characterised by effective communication and coordination systems: teams were aware of what was happening in the unit and could mobilise rapidly in response to emerging information or crises. Information regarding each woman was kept up to date and shared through well-functioning systems, including IT systems and/or whiteboards. Structured handovers, huddles and ward rounds allowed members of staff to have an accurate and readily available helicopter-level view of the whole unit. Conversely, poorly functioning information and coordination systems were widely recognised by participants as a significant threat to safety.

We have a really strong culture of doing regular ward rounds (…) so we’re not fire-fighting, we’re trying to anticipate what’s going to happen and where risk is. (Ethnography, interview with registrar, site 1)

We’re really strict with ward rounds, so every ward round there will be a multi-professional handover first of all (…) where all the clinical areas attend for that, including the neonatal unit, so that we can identify any risk factors there. So any potential ladies who might need to come to delivery suite or any ladies who have been here post-natally more than three days are always identified because they need a consultant review. (Ethnography, interview with midwife, site 2)

The moment they are free, people seem to gather in the office and look at the whiteboard and reassess what’s happening, and also write new information. Various people in the team, junior and senior, made sure that the whiteboard was legible and clear, for example one person deleted and rewrote everything using a different colour pen to improve legibility. It seemed to be a reliable and relied-on system. (Ethnographic observation, site 4)

Effective coordination was also achieved by tasking specific individuals with managing patient flow between the different care settings and safeguarding adequate staffing distribution. Together with reliable coordination systems, this allowed teams to respond to emergencies quickly and effectively.

The practice development midwife and the band 7 midwives did a lot of coordinating of staff, especially moving staff between antenatal, postnatal and delivery to plug staffing gaps, as well as keeping an eye on inductions and high risk pregnancies. The office of the band 7 midwives was very noticeable and central on the ward. And the door’s open all the time. (Ethnographic observation, site 2)

Finally, mandatory training emphasised the importance of situational awareness, which included enabling staff to recognise the important elements of their environment that may affect patient care. Consistent use of simulation-based training and structured emergency protocols allowed staff to be competent and confident in responding to crises.

Training in your role and in your unit is very [conducive] to make you understand what your role is. And to have the ability for the co-ordinator, senior obstetrician, registrar, but also for the healthcare support worker, to understand what the helicopter view of the room looks like, and actually to identify where the gap is. So if someone’s not writing, then you write, regardless of the fact that you’re a midwife. If that’s the gap, that’s what you do. (Ethnography, interview with midwife, site 1)

### The likely synergistic character of the features and the relevance of structural conditions

The features described in table 4 emerged through synthesis and thus take an idealised form which may not be present in full in individual units. Nonetheless, our cross-site analysis suggests that site 1 (the high-performing site) shows evidence of all seven features, while most other sites had some variations/combinations of the features. The seven features do not appear to act in isolation, each with independent effects; instead, every feature seems to interact with the next in a synergistic way, so what is seen at the level of the unit is the product of those interactions. No individual feature appears dispensable, and none on its own is sufficient. However, when all are present, they are likely to create a self-reinforcing cycle that promotes safety at a system level. The features of the framework might therefore be understood as synergistic. For example, in site 1, an emphasis on technical competence and shared expectation of high standards of practice enhanced mutual trust and positive working relationships and strengthened the team’s ability to mobilise quickly in an emergency. Commitment to safety was particularly strong because individuals’ contributions were noticed, acknowledged and used to make changes which, in turn, were then standardised and spread across the unit to enhance operational fit and avoid unwarranted variation. Importantly, this site showed a deep acknowledgement of the work required to preserve the ‘positive forces’ that characterised the unit.

Finally, our observations clearly indicated that inadequate structural conditions affected all the features described in the framework. Staffing and equipment shortages, for example, reduced teams’ ability to notice and react to small signs of safety deterioration, affected their capacity for debriefing, mentoring and informal knowledge sharing.

Staffing, the acuity of work. The complexities of women are so different (…) The health needs they’re presenting with, their co-morbidities. (…) The staffing issues… We’ve had peaks and troughs, we’ve had severe troughs. (…) Bed pressures are particularly bad… [It’s] a constant fight. (Ethnography, interview with midwife, site 1)

Not enough equipment. And, therefore, the equipment that we do get, gets used and used and used and used, and then breaks down, and then we've got less equipment. And, you know, it carries on and carries on. (Ethnography, interview with midwife, site 3)

## Discussion

Using data from hundreds of hours of observations, interviews and consultation, this study has produced a framework that summarises, describes and offers examples of the seven key features of maternity unit safety. In conducting this study, we aimed to deepen understanding of the features that make a maternity unit safe; we did not look for the features *unique* to high-performing units. Thus, we expect that most maternity units will show some combination of the features described in the framework (as did sites 2–6) and that the framework will help in identifying where units are already doing well and where further improvement efforts might usefully be targeted. This is critical because we hypothesise that the features of the framework may be synergistic: each feature is likely to act in concert with others, so that safety may be most optimised when all features are strong and mutually reinforcing. The For Us framework is intended to offer a plain language tool that aims to support those working in maternity units from any discipline or level of seniority to engage in reflection and learning, to identify and agree on priorities for improvement, celebrate achievements or to make a case for strengthening and investment.

In terms of high-level concepts, many of our findings are reassuringly consistent with those reported in other literatures that have looked at high performance, including high-reliability theory and resilience engineering. For instance, our analysis broadly aligns with the insight from resilience engineering that it is the people who work in organisations (in our example, maternity units) that constitute the major resource allowing systems to function effectively and flexibly.^23^ Our analysis also aligns with many concepts from the high-reliability literature, such as attention to failures and learning, the importance of socialising processes, climates of trust and respect, the quest for constant improvement, vigilant coordination of upstream and downstream work, high-quality operations and human resource management practices and fostering collective awareness and alertness.^43 44^

The particular strength of our analysis is to make vivid and relevant the features of safety for maternity units, thus making clear ‘what good looks like’ for maternity care specifically and providing clear directions for practice through a plain language framework. It thus has much in common with the increasingly influential field of positive deviance, with its emphasis on specificity and actionability.^26 27^ The framework we produced does not itself mandate improvement actions or interventions. This absence is deliberate: it is intended to place the authority and agency for improvement with those who provide care and those whose role it is to support them.

The methodological approach we have used here, in particular its use of ethnography, has similarities with those used initially in the development of high-reliability theory.^44^ Use of a multisite approach significantly deepened and extended the analysis yielded by study of one site only.^18^ The additional use of stakeholder consultation was valuable in ensuring clarity and comprehensiveness of the framework and relevance to the intended user group. The approach and our findings complement previous qualitative research that has examined features of maternity care systems that might affect care in different birth settings (eg, obstetric units and freestanding midwifery units).^45^

Our study does have some limitations. First, only the index site was chosen on the basis of its exceptional performance, and it is possible that more could have been learnt about high performance through inclusion of further examples of very high performance—though this might also have limited other opportunities for learning. Second, data in the index site and in the rest of the sites were collected by the same researchers, meaning that the learning from the first phase of the ethnography might have influenced data collection and analysis in the second. We sought to contain this problem by tasking researchers who were not involved in the first phase with the analysis of samples of transcripts from sites 2–6 and with noticing any data that did not seem to fit with the pre-existing framework. Third, in its focus on safety, For Us does not cover other important aspects of quality of maternity care, such as antenatal care continuity or families’ experiences. Fourth, For Us is currently applicable to hospital-based maternity services only; more evidence is needed to establish whether the framework applies to community-based services or other care settings and outside of a UK context.

The For Us framework focuses mainly on culture, behaviours and processes and thus illuminates the relatively low-resource changes that might make a difference for patient safety but might otherwise remain invisible or be regarded as ‘fluffy’. These are important targets for intervention, especially as evidence accumulates of their tractability to purposeful improvement efforts.^46 47^ However, it is important not to underplay the impact of structural factors on safety (including staffing levels, quality and availability of equipment and physical environment). Future work should also identify how best to implement this framework and to evaluate how far optimal implementation results in improved outcomes.

## Data Availability

Our ethical approval does not allow sharing of primary data. Requests for data should be directed to Mary Dixon-Woods (PI for the project).
