# Supplementary material for: Seven features of safety in maternity units: a framework based on multisite ethnography and stakeholder consultation
Source: BMJ Qual Saf. 2020 Sep 25;30(6):444–56. doi: 10.1136/bmjqs-2020-010988 (PMC8142434; doi:10.1136/bmjqs-2020-010988)
Supplement: Supplementary data [file bmjqs-2020-010988supp001.pdf]

Supplementary file 1

For Us Framework development process, explained

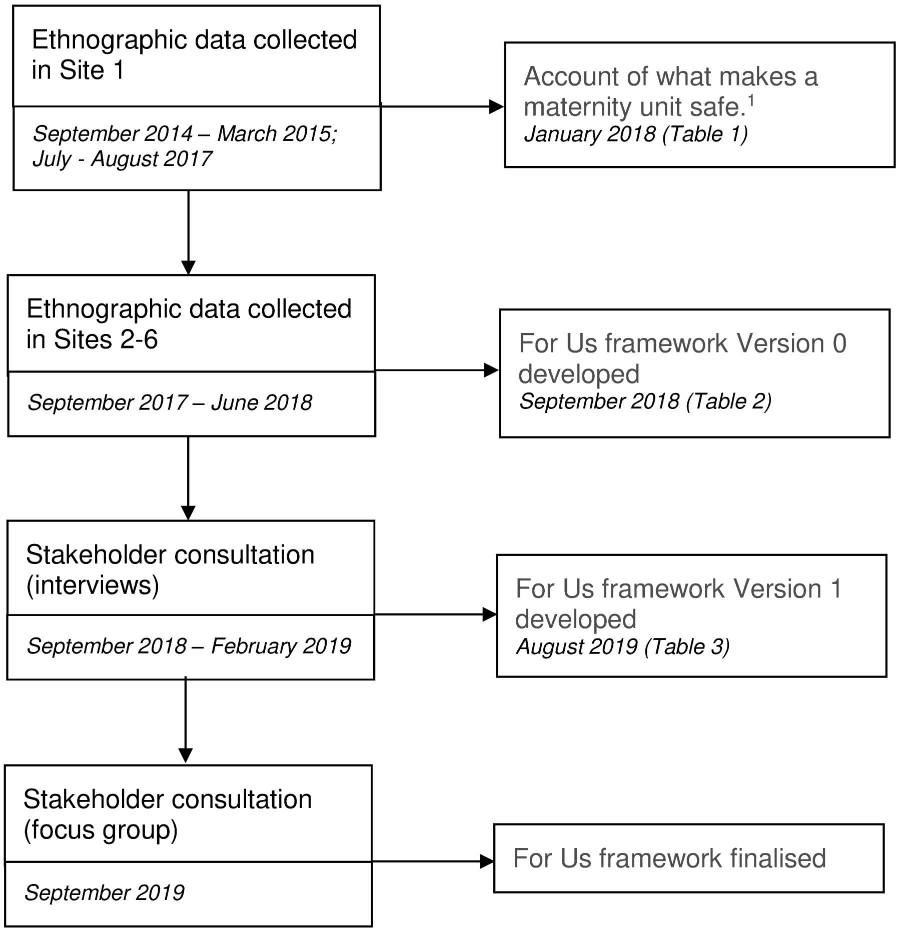

UNIVERSITY OF CAMBRIDGE

Account of what makes a maternity unit safe (January 2018)

Table 1: Theoretically informed account of what makes a maternity unit safe, as reported Liberati et al, 2019.[1] Data sources: ethnographic data collected in Site 1.

Mechanism	Observable indicators
1. Collective competence	• Interdependency, collegial behaviours, and strong social ties among staff<br>• Mutual respect across roles and disciplines<br>• Care organised around the shared goal of safe childbirth, with professional boundaries managed flexibly<br>• Sapiential authority: deference to expertise rather than hierarchy
2. Insistence on technical proficiency	• Expectation of very high standards of proficiency in clinical tasks<br>• High-fidelity, realistic training to develop technical competence<br>• Informal training and role modelling in routine care delivery (e.g. clinical cases discussed during handovers or informal conversations)
3. Monitoring, coordination, and distributed cognition	• Mechanisms and roles allocated to maintaining a shared awareness of the external situation in the maternity unit<br>• Staff in coordinating roles playing a control room function<br>• Constant effort to ensure that the team is fit to cope with the circumstances
4. Clearly articulated and constantly reinforced standards of practice, behaviour, and ethics	• Values and standards are clear, articulated, and reinforced through role modelling<br>• Sense of discipline and a lack of tolerance of cutting corners<br>• Social control: individuals take actions to ensure that other people behave in a way that is aligned with the unit's standards
5. Monitoring multiple sources of intelligence about the unit's state of safety	• Data are used to sense problems<br>• Hard indicators: routine clinical data are constantly scrutinised, updated, and made available to all staff<br>• Soft intelligence: use of patient complaints and staff ground knowledge to learn and improve safety<br>• Psychological safety: staff can raise safety concerns without fear of embarrassment, retaliation, or punishment
6. Highly intentional approach to safety and improvement	• Commitment towards safety is collectively pursued and socially legitimised (not externally imposed)<br>• Organisational citizenship behaviours: discretionary effort to promote the safety and effective functioning of the unit<br>• Combination of formal risk management (i.e. allocated roles and formal activities, such as safety checks) and embedded risk management (frontline clinicians proactively preparing for risky situations and detecting small signs of deterioration)

For Us framework Version 0 (September 2018)

Table 2: First iteration of a plain language framework of features of safe maternity units. Data sources: whole ethnographic dataset (with examples from all six sites).

Features of safe maternity units	Description and examples
**Staff in the unit have good relationships, cooperate effectively, and work as a team**	• Teamwork is central to all of the unit's activities. Care delivery, training, research, and risk management are conducted with the input of all relevant professions and medical specialties.<br>• Lone ranger behaviours are discouraged.<br>• Midwives and doctors value each other's contributions; disagreements are settled through open discussion rather than resorting to hierarchies or power.<br>• People are aware of each other's roles, skills, and competencies (who does what, how, and when).<br>• There is an expectation that the person with more experience and the right skills (rather than more seniority) will take action when needed.
**Staff perform their clinical tasks competently**	• Staff members are very technically competent in performing their clinical tasks and show confidence and calmness in high-stress situations (e.g. emergencies).<br>• Formal training sessions (e.g. skill drills and simulations) are available in the unit to enable staff to develop their technical and specialised skills.<br>• Formal training is not the only learning modality: people also learn through observing colleagues and discussing clinical cases informally during work.
**Colleagues communicate effectively, activities are carefully coordinated, and people know what to do in an emergency**	• Systems and processes are in place to maintain situational awareness, which means that staff recognise and understand the important elements of their environment.<br>• Regular handovers, ward rounds and board rounds are conducted. A whiteboard (or a similar aide) is used to capture and share up-to-date information regarding all the women admitted to the unit.<br>• There are communications systems and processes in place, as well as specific professional roles to secure communication, coordination, and effective patient flow between the different care settings.
**The rules and norms relating to how people should behave are clear, and people intervene if and when these are broken**	• There is a sense of discipline and intolerance of cutting corners. For example:<br>○ making mistakes is tolerated but hiding them is not<br>○ safeguarding the dignity, safety, and psychological wellbeing of women admitted into the unit is paramount.<br>• The values and rules are not only made explicit but also lived and enacted: they are reinforced through role modelling, both by peers and by senior individuals.<br>• Individuals take actions to ensure that colleagues behave in an appropriate way. They may use informal ways to bring behaviour back in line when needed (e.g. humour, or having a private word), but are prepared to intervene more formally (e.g. formal reporting or escalating to senior staff) when needed.
**The unit collects multiple types of data (including feedback from pregnant women and frontline staff), and use them to improve safety**	• Clinically relevant data are collected and examined to identify concerning trends and guide improvement efforts.<br>• Data are used to sense problems (as opposed to seeking comfort).<br>• Women's or parents' complaints are taken seriously and routinely investigated, and may be used in training contexts.<br>• Staff are carefully listened to. They can raise safety concerns, with confidence that the team would not embarrass or punish them for speaking up or disagreeing.
**Staff are personally committed to improving safety and the way the unit works, and this results in efficient and effective working processes**	• Frontline staff are constantly at work to fix problems and make small changes to improve the safety of the unit, driven by their personal commitment to delivery safe care<br>• Commitment to safety is not externally imposed: it is value-driven and embedded in group identity<br>• Staff use learning from risky situations or adverse events to self-correct and improve the management of risks<br>• The unit has a range of risk management systems, processes and roles (risk management team, audits, safety checks, etc.), that people are aware of<br>• The physical environment of the unit, its infrastructure and equipment are carefully designed for the tasks that teams need to conduct

Liberati EG, *et al. BMJ Qual Saf* 2021; 30:444–456. doi: 10.1136/bmjqs-2020-010988

For Us Framework Version 1 (August 2019)

Table 3: Second iteration of the For Us framework. Data sources: ethnographic dataset; stakeholder consultation (65 interviews).

Features of safe maternity units	Description and examples
**Authentic commitment to safety and improvement at all levels, with everyone involved**<br><br>**Frontline staff, management, and the unit as a whole show a commitment to safety and to continuous improvement.**	• The unit is authentically committed to learning from risky situations and adverse events, and it uses this learning to drive improvements.<br>• Staff are highly alert to hazards and are skilled in noticing them. They seek to address threats to safety in real-time and are careful to report them, so that the whole unit can learn.<br>• Staff invest in making the unit better. They are always looking for ways to improve working processes and the care environment – often through small-scale, easily actionable ideas – and are praised for their efforts.<br>• Individuals in management roles are highly visible and accessible. They listen carefully to frontline staff, women, and their families, seeking to respond promptly to concerns or suggestions reported to them.<br>• The unit has a range of formal risk management systems, processes, and roles (including audits and/or a risk management team) that are known, trusted and used by staff in the unit.
**Insistence on technical competence, supported by formal training and informal learning**<br><br>**Staff are educated and trained, through multiple methods, to perform their tasks to a very high standard of competence.**	• Individuals are expected to perform their clinical tasks to a very high standard of proficiency.<br>• The unit invests in keeping staff trained and up to date.<br>• Regular high quality training sessions (including structured teaching, skill drills, and simulations) are mandatory for all members of staff, and the unit management ensures that everyone has allocated time to attend.<br>• People also learn in less formal ways, for example through mentorship, observing colleagues at work, and discussing and reflecting on clinical cases.<br>• A social space is accessible to all staff (a communal coffee room, for example) to support informal knowledge-sharing, real-time information updates, and reflection.<br>• Senior members of staff make sure that junior staff have opportunities to debrief after experiencing complex clinical situations, and that they learn from theirs and others' experience.<br>• The many different forms of education and socialisation allow staff to demonstrate competence, confidence, and coordination in high-stress, risky situations, and help to create trust among team members.
**Teamwork, cooperation, and positive working relationships**<br><br>**Staff have good relationships, understand each other's roles, and work effectively across professional boundaries.**	• Teamwork is central to all of the activities carried out in the unit. Care, training, and research are conducted with the input of all professions and disciplines.<br>• People in different roles respect each other and value everyone's contributions to achieving the goals of the unit and upholding its values.<br>• Through working and training together, people are aware of each other's roles, skills, and competencies (who does what, how, why and when) and can work effectively together, thus demonstrating "collective competence".<br>• There is an expectation that the person with most experience and the right skills (not necessarily the most seniority or a particular professional role) will take the actions needed for high quality clinical care. Arguments about professional boundaries are absent or are resolved through reference to shared goals.<br>• When disagreements happen between professions or roles (for example on treatment decisions), they are settled calmly through open, thoughtful discussion rather than resorting to hierarchies, displays of power, or aggressive behaviour.<br>• People look after each other. Relationships are good, and any disruptive or bullying behaviours are recognised and controlled effectively.<br>• Staff wellbeing and morale are recognised as important contributors to safety.
**Constant reinforcing of safe, ethical, and respectful behaviours**<br><br>**Through role-modelling, leading by example, and mutual monitoring, staff ensure that everyone in the unit behaves according to the expected standards of practice.**	• The goals and values of the unit are clear: achieving good birth outcomes and promoting the dignity and wellbeing of women and babies, supported by evidence-based practices.<br>• There is a shared expectation that people will behave consistently in line with these goals and values, and newcomers are actively socialised into the unit's norms.<br>• Expected standards of practice are reinforced through the behaviours of everyone in the unit, including all professions and individuals at all levels – from the most junior to the most senior.<br>• People intervene if the goals and values of the unit are not upheld. They do so mostly in informal ways (for example by using humour or having a 'private word'), but are ready to intervene more formally (for example through reporting systems and escalating) when needed.<br>• Unsafe or inappropriate behaviours are noticed and corrected in real time, so they don't become normalised.<br>• Although the highest standards of practice are expected, it is recognised that errors may sometimes happen.<br>• Errors are recognised both as problems and as opportunities for learning. People are encouraged to discuss them openly, and actions are taken to reduce risk of their recurrence.

Liberati EG, *et al. BMJ Qual Saf* 2021; 30:444–456. doi: 10.1136/bmjqs-2020-010988

**Multiple problem-sensing systems, used as basis of action**<br><br>**Women on the unit and members of staff are actively encouraged to speak up and carefully listened to. Their voices, as well as clinical data, are used to sense problems, self-assess, and learn.**	• The unit uses multiple methods to 'sense' problems and identify opportunities for improvement, including staff and patient voice, hard data, and clinical simulation.<br>• The multiple forms of intelligence are also used to identify good practices and celebrate them where appropriate.<br>• Women and families are encouraged to share their experience, both informally and through formal feedback systems. This feedback is seen as key intelligence for improving care.<br>• Members of staff feel that they can speak up for safety. They are confident their concerns will be heard and that action will, if possible, be taken as a result.<br>• This sense of psychological safety cultivated on the unit makes it possible to learn from everyday events.<br>• Clinically relevant data are collected and constantly monitored using visual methods (a clinical dashboard, for example) to identify concerning trends and guide improvement efforts.<br>• Members of staff are reminded about the importance of looking at and interrogating data.
**Systems and processes designed for safety, and regularly reviewed and optimised**<br><br>**The unit's physical environment, systems, and processes are continually improved to ensure usability and operational fitness.**	• Working processes and information technology are carefully designed, and kept functional and up to date.<br>• The unit's equipment and the physical environment are designed consistent with human factors principles to be safe, appropriate, and easy to use.<br>• People constantly review and seek to optimise working processes (operating theatre scheduling, for example) and tools (post-partum haemorrhage kits, for example) to meet the requirements of excellent care provision.<br>• Simulation is used observe how systems and processes operate in realistic conditions and to test the usability and appropriateness of equipment and other resources needed for care.<br>• Once good practice is identified, it is standardised and spread across the unit, to avoid unwarranted variation.
**Effective coordination and ability to mobilise quickly**<br><br>**Through effective communication and coordination systems, teams are aware of what is happening in the unit, and can mobilise rapidly in response to emerging information or crises.**	• Well-functioning systems (including IT systems and/or whiteboards, for example) are in place to capture and share up-to-date information regarding each woman.<br>• These systems help to notice early signs of safety deteriorating, and to quickly initiate an effective response.<br>• Regular handovers, ward rounds, and board rounds enable a shared, helicopter-level understanding of the state of the unit as a whole, in real time.<br>• Identified individuals in the team (for example the midwife coordinator) have specific responsibility and expertise for patient flow and management between the different care settings.<br>• Mandatory training emphasises the importance of situational awareness, which includes enabling staff to recognise the important elements of their environment that may affect patient care.<br>• Staff are highly competent and confident in responding to emergencies, including rare events, supported by simulation-based training.

1. Liberati EG, Tarrant C, Willars J, et al. How to be a very safe maternity unit: An ethnographic study. Soc Sci 2019; 223:64–72.
